# Optimising Extinction of Conditioned Disgust

**DOI:** 10.1371/journal.pone.0148626

**Published:** 2016-02-05

**Authors:** Renske C. Bosman, Charmaine Borg, Peter J. de Jong

**Affiliations:** Department of Clinical Psychology and Experimental Psychopathology, University of Groningen, Groningen, the Netherlands; Technion—Israel Institute of Technology, ISRAEL

## Abstract

Maladaptive disgust responses are tenacious and resistant to exposure-based interventions. In a similar vein, laboratory studies have shown that conditioned disgust is relatively insensitive to Conditioned Stimulus (CS)-only extinction procedures. The relatively strong resistance to extinction might be explained by disgust’s adaptive function to motivate avoidance from contamination threats (pathogens) that cannot be readily detected and are invisible to the naked eye. Therefore, the mere visual presentation of unreinforced disgust eliciting stimuli might not be sufficient to correct a previously acquired threat value of the CS+. Following this, the current study tested whether the efficacy of CS-only exposure can be improved by providing additional safety information about the CS+. For the CSs we included two neutral items a pea soup and a sausage roll, whereas for the Unconditioned Stimulus (US) we used one video clip of a woman vomiting and a neutral one about glass blowing. The additional safety information was conveyed by allowing actual contact with the CS+ or by observing an actress eating the food items representing the CS+. When additional safety information was provided via allowing direct contact with the CS+, there was a relatively strong post-extinction increase in participants’ willingness-to-eat the CS+. This beneficial effect was still evident at one-week follow up. Also self-reported disgust was lower at one-week follow up when additional safety information was provided. The current findings help explain why disgust is relatively insensitive to CS-only extinction procedures, and provide helpful starting points to improve interventions that are aimed to reduce distress in disgust-related psychopathology.

## Introduction

From an adaptive stance it has been argued that disgust evolved with the core function of protecting individuals against contamination by facilitating avoidance of toxins and pathogens [1, 2]. Accordingly, disgust has been conceptualised as a disease-avoidance mechanism [3]. In spite of this functional role, there is also increasing evidence implicating disgust in the aetiology and maintenance of various types of psychopathology [4]. In line with this, disgust often complements fear as a common feature of specific phobias such as spider phobia [5–7], emetophobia [8], and blood-injection-injury phobia (BII) [9]. In addition, disgust is a frequent symptom of post-traumatic stress disorder (PTSD) [10], and contamination-based obsessive-compulsive disorders (C-OCD) [11–12].

There is ample evidence that in vivo exposure is highly effective in reducing dysfunctional fears [13]. Although several studies showed that exposure based interventions may also weaken disgust [5], the decline of disgust has typically been shown to be slower than that of fear [14–15]. In other words, it seems that exposure in vivo treatment is less effective in reducing disgust compared to fear. In a similar vein, experimentally conditioned disgust responses (CRs) have also been shown to be relatively insensitive to extinction procedures [16–18]. Specifically it has been found, that although the strength of subjective disgust does show some decline following a series of Conditioned Stimulus (CS)-only presentations, even after an extensive series of extinction trials differential disgust responding to the CS+ vs. CS- typically remains [16, 19]. Based on these types of findings, disgust conditioning has been described as a sticky form of relational learning that is resistant to extinction [20]. Thus a critical question is how to explain the apparent differential sensitivity of acquired fear vs. acquired disgust to traditional extinction procedures: why does conditioned disgust persist where fear typically subsides following CS-only extinction procedures (for review see, [21]). Answering this question seems especially pressing in light of the accumulating evidence suggesting that residual disgust following treatment may contribute to relapse of disorders such as specific phobias and PTSD [16].

In the typical fear-conditioning paradigm, electro-cutaneous stimulation is used as the aversive outcome (e.g., [22]). This type of US seems a proper prototype for a threat of getting physically harmed (e.g., bitten by a dog or stalked/touched with a knife). Importantly, the sources of these types of threats are visible and the absence or presence of the threatening outcome (e.g., a bite) can be readily noticed: you do or do not experience pain. Clearly then, a series of CS-only presentations would be sufficient to inform the participant that the CS is no longer a signal of impending threat, thereby removing the source of fear.

However, disgust serves an important function in countering qualitatively different but equally relevant threats (i.e., contamination by pathogens/toxins) that cannot be readily detected, and the sources of these threats cannot be seen by the naked eye [1]. As an example, a person inflicted with a sexually transmitted infection can be highly contaminating, also in the absence of obvious symptoms or cues that signal the person’s contaminating properties. Because it is difficult to decide whether a stimulus is free from pathogens by mere visual inspection, CS-only presentations may not provide the type of safety information required/necessary to undermine the threat-value of the CS+, and thereby to remove its properties to elicit a disgust response.

Although it is proposed that the ultimate factor why people may quickly learn disgust is prevention of contamination [1], this does not imply that people feel disgust and therefore avoid the CS+ because they think it might be contaminating; at the proximal level, individuals just experience disgust and aversion towards the CS+. However, the perspective that disgust evolved as a disease avoidance mechanism does imply that to counteract the acquired disgust it requires compelling information confirming that the CS+ is in fact safe to approach. Accordingly, it seems plausible to assume that just being repeatedly visually exposed to a disgusting item may not be sufficient to provide the necessary safety information to change its signal (threat) value. In line with this, in their seminal review Rozin and Fallon argued that intense physical contact is an important pathway to reduce disgust [23].

Supporting the relevance of actual physical contact in reducing acquired disgust, a recent disgust-conditioning study—using food items as CSs (i.e., cheese and a bruschetta wrap) and the same US material we use in the current study—showed that the extinction of conditioned disgust was most pronounced for participants who were exposed to a Behavioural Approach Task (BAT) before the actual extinction procedure (i.e., CS-only presentations) [18]. This finding highlights the relevance to more systematically test the hypothesis that extinction of conditioned disgust can be potentiated by providing more convincing information about the safety of the CS+ than is usually provided by CS-only presentations.

Thus the first and major aim of the current study is to test if indeed the efficacy of CS-only exposure can be improved by somehow providing information that the CS+ is safe to approach. Following the approach of Borg et al. [18], we used food items as the CS+/CS-. We tested the efficacy of two pathways to convey safety information about the CS+. As a first strategy we again exposed part of the participants to a BAT before the start of the extinction procedure. Second, we exposed part of the participants to a video-clip in which an actress was texting on her mobile phone while simultaneously eating from the food items representing the CS+. To control for the effect of mere exposure time to the CS+/CS- items, the duration of both conditions was the same. To specifically test whether indeed (showing) the apparently safe ingestion of the CS+ was critical, we included a control condition in which an actress was similarly involved in texting but in this clip she did not eat (any of) the food items. As a subsidiary aim, we tested the robustness of the effects of the current extinction procedures. Therefore, we tested both the immediate and the longer-term effects of providing additional safety information on participants’ disgust responding to the CS+/CS-.

In this study we did not only rely on self-report measures but also included facial electromyography (EMG) as a more implicit index of disgust (un)learning [24]. This seems especially relevant as previous research mainly relied on self-report indices of disgust, whereas the scarce studies that did include facial EMG as the physiological marker of disgust failed to show a comparable pattern for both types of indices [18, 25]. Thus the third aim of this study was to explore whether the impact of safety information on the CR might be restricted to the subjective level or would also be evident at the physiological level.

All in all, the current experiment is aimed at testing: i) the efficacy of providing additional safety information on weakening the disgust-relevant threat value attached to the CS+ when compared to the traditional CS-only exposure, ii) the long-term effect of providing such information; and iii) whether the impact of this additional information on the CR would be translated to the physiological level or be restricted to self-reports.

## Method

### Participants

Participants were first year female psychology students [N = 133, M_age_ = 20.1, SD = 2.8] who were recruited via an internal university system. From the initially recruited sample of 142 participants, nine participants were excluded post hoc due to poor compliance, dislike for one of the food items, or problems with data acquisition. The Dutch Ethical Committee of Psychology (ECP) at the University of Groningen approved this study. Specifically, all participants gave their written informed consent and all procedures of the study (including the written informed consent, and the autonomy of each participant to stop at any point during the study) were kept in line with the ECP regulations. No minors (<18 years of age) were included for this study.

### Materials

#### Stimuli

The Conditioned Stimuli (CSs) consisted of two types of neutral food items–a pea soup and a sausage roll, each pictured from two different angles (i.e., 2 pictures of the two mentioned food items).

As the disgust-relevant Unconditioned Stimulus (US), we used a clip depicting a woman vomiting [6, 26]. As a neutral outcome we used a neutral clip depicted the making of handmade glass [26]. Both types of clips consisted of 8 5 seconds sequential film excerpts, together forming a storyline of 40s. Both clips were presented with sound, so that if participants would look away from the clip they would still know what was happening.

#### Validation

For selecting the optimal stimuli, a validation test of the CSs and USs was done prior the experiment [N = 41 women, Mean (M)_Age_ = 20.0, Standard Deviation (SD) = 2.2 years]. For the CSs, we used visual analogue scales (VAS) to measure ‘disgust’ (0 = not at all disgusting, 100 = very disgusting), ‘willingness-to-eat’ (0 = not at all willing, 100 = very willing), and ‘valence’ (0 = negative, 100 = positive). Both items used as CS were considered neutral (i.e. rated between 40–60 on all of the three VAS-dimensions) and were not significantly different from each other.

The US_Disgust_ and US_Neutral_ clip were validated on the dimension of ‘disgust’ and ‘valence’. In line with expectations, on the dimension ‘disgust’, the US_Disgust_ was rated as highly disgusting [M = 99.5, SD = 1.6], while the US_Neutral_ was rated as very low on disgust [M = 5.8, SD = 11.4, F (1, 80) = 2705.1, p < 0.001, $\eta_{p}^{2}$ = 0.97]. Similarly, on the dimension of ‘valence’, the US_Disgust_ was clearly rated as more negative with a mean score of 1.0, SD = 3.2, while the US_Neutral_ was rated more positively [M = 77.8, SD = 20.7, F (1, 80) = 552.7, p < 0.001, $\eta_{p}^{2}$ = 0.87].

### Measures

#### Self-reports

The Disgust Propensity and Sensitivity Scale Revised (DPSS-R) [27] is a 12-item measure of disgust propensity (i.e., how frequently someone feels disgusted) and disgust sensitivity (i.e., how negative someone interprets the feeling of disgust). In this study Cronbach’s α for the DPSS-R propensity scale was 0.69 and 0.70 for the sensitivity scale.

The Vancouver Obsessive Compulsive Inventory—Contamination Fear Subscale (VOCI) [28–29] is a 12-item measure of contamination obsessions and washing compulsions. The Cronbach’s α for the subscale was 0.89 in the present study.

The Hunger Scale (HS) [30] is a 4-item questionnaire that measures how much participants could eat of their favourite food, and the time passed from their last meal. In this study we used one item from the HS, i.e., ‘How hungry are you at this moment?’.

Visual Analogue Scales (VASs) were used to index the subjective appreciation of the CSs; we assessed three relevant dimensions: disgust (0 = not at all disgusting, 100 = very disgusting), willingness-to-eat (0 = not at all willing, 100 = very willing), and in line with previous fear-conditioning research [31] we finally measured the more general affective valence elicited by the CS (0 = negative, 100 = positive).

#### Facial EMG

Electromyographical (EMG) activity was measured with PortiLab2 (hardware: Porti5-16/ASD) with pairs of Ag-AgCI electrodes (diameter: 6 mm), placed on the left side of the face according to standard recommendations [32]. Data were recorded from the levator alesque nasii muscle (levator) as a unique marker of disgust relative to other negative emotions. Additionally, the musculus corrugator supercilii (corrugator) was selected as an index of general negative emotions [33–34]. Fz functioned as the reference point and the EMG-signal was sampled at 2000 Hz.

#### Approach behaviour

A Behavioural Approach Task (BAT) was used to assess the actual approach tendencies towards the CS+ and CS-. The CS+ and CS- food items were placed on separate plates that were covered and numbered. Participants were asked to uncover the indicated plate and take a bite of the food item (step 1). In step 2 participants indicated whether they had indeed eaten from the food item on a binary scale. The order of presenting the CS+ and CS- food-items was randomised.

### Conditions

To test the value of providing additional safety information participants were split into four different conditions: (1) acquisition was directly followed by the extinction procedure (n = 35, control/*no-exposure condition*); (2) acquisition was followed by a BAT before moving on to extinction (n = 33, *BAT condition*, approx. 220s); (3) acquisition was followed by the presentation of a film clip displaying explicit safety information about the CS+ and CS- (i.e., a female student eating the CS+/CS- and concurrently having the other food item being exposed whilst using her phone, n = 33, *active enforcer condition* (220s); (4) just as in condition 3 acquisition was followed by a film clip (220s) depicting the CS+ and CS- together with the same actress using her phone, yet in this clip she did not eat or touch the CS+/CS- food items. Thus this condition controlled for the mere presentation of the CS+/CS- without providing explicit safety information (n = 32, *inactive enforcer condition*).

### Procedure

Upon arrival at the laboratory, participants were provided with written information about the study and were asked to read and sign the informed consent. Participants started with some demographic questions and completed the HS. Then the EMG electrodes were attached. Beforehand, participants were assisted to make themselves as comfortable as possible to reduce unnecessary movement and distraction. The skin was cleaned with NuPrep scrub to optimise the conduction of the EMG signal, and the electrodes were filled with conductivity gel. Participants were asked to remain focused on the computer screen during the whole task. During the entire experiment, participants could communicate with the researcher via a microphone. The task was separated into four phases. After the computer task, participants completed the HS again and also completed the other questionnaires.

During phase 1 (*habituation*) participants were familiarised with the four CS pictures (i.e., 2 types of food stimuli, each presented from two different angles). Each CS-picture was presented for 6 seconds, followed by VAS ratings for baseline measurements. After each CS an inter trial interval (ITI) followed which had a mean duration of 10 seconds, fluctuating between 8–12 seconds. During the ITI a fixation cross (+) was presented on the screen. Throughout the computer task, the ITI always had the same characteristics.

During phase 2 (*acquisition*) participants learned the CS-US contingencies. The CS+ was always followed by the US_Disgust_, whereas the CS- was always followed by the US_Neutral_. A trial consisted of 8 CS-US pairs in which each CS lasted 6 seconds and each US 5 seconds. The 40 seconds of US formed one storyline. A trial ended with an ITI. Both types of trials were presented 10 times, resulting in 20 trials with a total duration of 37 minutes. The order of the neutral and the disgust trials was random. After the last trial participants rated the CSs again on the same VASs as were used in habituation. In phase 3 (*exposure)*, the sample was split into the four different conditions (see Conditions). For all conditions, except the *no-exposure control condition*, the CSs were again rated after the manipulation on the same VASs (see Measures).

Phase 4 (*extinction*) was the same for all participants. The CSs were presented for 6 seconds and followed by an ITI. Each CS picture was presented 10 times, resulting in 40 trials. Following the 15-minute extinction phase, participants again rated the CSs on the VASs and subsequently completed the post-extinction BAT. Following the BAT, electrodes were removed before participants were asked to complete the questionnaires. The laboratory session was concluded with a (short) debriefing in which participants were asked to check their e-mail for the follow up assessments 24 hours and one week after the lab session. This e-mail provided participants with instructions and a link to the online measurement.

To assess the longer-term impact of the conditioning procedures, participants rated the CS+ and CS- pictures again at (approximately) 24 hours and 7 days follow up. This was done via the internet using ‘Qualtrics’ platform (Qualtrics, Provo, UT). To disguise the actual aim of the assessment, the CS+ and CS- pictures were presented intermixed with two other food pictures (used as CSs in a previous conditioning study, a bruschetta and a block of cheese) [18]. After completion, participants received via e-mail a written debriefing about the nature of the study.

### Data reduction and analysis

All data were analysed with SPSS (version 20.0.0) using a 5% level of alpha to test for significance. To check if the ratings of the food items for each VAS separately were comparable to the outcomes of the validation study a one-way ANOVA was used. To test for pre-existing group differences that can influence disgust learning we used a number of questionnaires (see method): For the DPSS-R, we computed two subscales i.e., disgust propensity and disgust sensitivity. For the VOCI we used the total sum of all the questionnaire items. We used one item from the HS to measure the levels of hunger.

The EMG data were processed offline and filtered (high-pass: 10 Hz; low-pass: 500 Hz). To ensure the quality of the data we visually inspected each trial within Aphys (version 2.1.2.0) [35] before being accepted; missing trials or trials containing artefacts were replaced with the mean (root-mean-square) EMG activity. The Aphys program calculates mean levels of activation for predefined epochs. For the current analyses Aphys provided indices of mean EMG activation during the pertinent stimuli (CS-slide or US-clip) by comparing mean EMG activity during a 100ms baseline epoch immediately preceding a particular stimulus and the mean EMG activity during the actual stimulus presentation. Thus each stimulus presentation had its own 100ms baseline thereby controlling for overall changes in the EMG signal (e.g., drifts) unrelated to responses of participants to the pertinent stimuli. Due to the atypical set-up used in acquisition, it was impossible to have a neutral baseline interval for all of the presented stimuli; in line with our previous study [18]—we therefore only used the first CS presented in each of the 10 acquisition trials. To further reduce the impact of noise/artefacts and to enhance the reliability of our EMG indices, for each of the relevant stimuli (CS+, CS-, US_Disgust_, US_Neutral_) we relied on the mean EMG activation across trials during the various stages of the experiment [18]. To index EMG responding during habituation we computed mean EMG activity during all relevant trials of habituation and the first trial of acquisition (given that participants were at that stage still naive about the US). Mean EMG activity during acquisition was based on all of the remaining acquisition trials. Mean EMG activity during extinction was based on all pertinent trials of the CS-only extinction stage. In the pre-processing stage Cook’s distance was calculated for all variables and used to check for (influential) outliers. Since for all variables yielded that Cook’s distances <1, no further corrections were carried out.

To first test whether the current design was successful in learning disgust, VAS data were subjected to a 2 CS (CS-, CS+) * 2 Phase (habituation, acquisition) repeated measure analysis of variance (RM-ANOVA) with both factors being within subjects factors. In addition, EMG data were analysed via a 2 CS (CS-, CS+) * 2 Muscle (levator, corrugator) * 2 Phase (habituation, acquisition) multivariate RM-ANOVA.

Second, we tested whether disgust is sensitive to extinction via CS-only exposure or whether offering more safety information as an additional exposure strategy would enhance the sensitivity to the extinction of the newly learnt disgust. Therefore, VAS scores were subjected to 2 CS (CS+, CS-) * 2 Phase (acquisition, extinction) * 4 Condition (no-exposure, BAT, active enforcer, and inactive enforcer) with only the latter factor being a between subjects factor. In addition, EMG data were analysed with a 2 CS (CS-, CS+) * 2 Muscle (corrugator, levator) * 2 Phase (acquisition, extinction) * 4 Condition (no-exposure, BAT, active enforcer, inactive enforcer) multivariate RM-ANOVA. For the long-term measurements VAS scores were similarly subjected to 2 CS (CS+, CS-) * 3 Phase (extinction, post 24hours, post 7 days) * 4 Condition (no-exposure, BAT, active enforcer, and inactive enforcer) RM-ANOVA. To further decompose interaction effects, paired t-tests were used. When in these analyses Mauchly’s test of sphericity was found to be significant, Greenhouse-Geisser adjusted degrees of freedom were used. Finally, to assess whether there were significant differences between conditions in the actual approach (as measured by the task to take a bite of the actual food items), we calculated the percentages of participants eating the CS+, the CS- and participants not eating any of the two food items, and subjected these scores to *X*^*2*^ test analysis.

## Results

### Manipulation checks

#### Pre-existing condition differences

Given that there are no indications of condition differences for the level of hunger as measured by the HS [F (3, 129) = 1.26, p = 0.29], further analysis of this questionnaire is collapsed over conditions. Participants did not change their level of hunger from the start (i.e. the first time the HS was administered) [Mean (M) = 3.0, Standard Deviation (SD) = 1.6] to the end of the experiment (i.e. the second time the HS was administered) [M = 3.1, SD = 1.8; *t* (131) = 0.64, p = 0.53]. Thus, throughout the experiment the hunger level remained virtually the same. In addition, there were no indications of condition differences with regards to trait disgust propensity and trait disgust sensitivity [Propensity: F (3, 129) = 2.1, p = 0.11; Sensitivity = F (3, 129) = 1.0, p = 0.38]. The overall mean for disgust propensity was 13.6 [SD = 3.0] and 7.8 [SD = 3.5] for disgust sensitivity. Yet, there were pre-existing group differences with regard to the scores on the VOCI [F (3, 129) = 3.3, p = 0.02]. Post-hoc analysis showed that participants in the inactive enforcer condition had higher ratings on the VOCI [M = 10.7, SD = 9.1] than those in the BAT condition [M = 5.2, SD = 4.6; Post-hoc LSD: M_dif_ = 5.5, p = 0.002]. There were no differences between any of the other conditions [p’s ≥ .06].

### Disgust learning

#### Was disgust successfully learned as evidenced by the subjective ratings?

In S1 Table the Ms and SDs are provided as a function of experimental Phase (habituation, acquisition, extinction, post 24 hours, post 7 days), CS (CS+, CS-) and Condition (no-exposure [control condition], BAT, active enforcer, inactive enforcer). Manipulation checks for the conditioned stimuli can be found on S1 Appendix.

**Fig 1:**
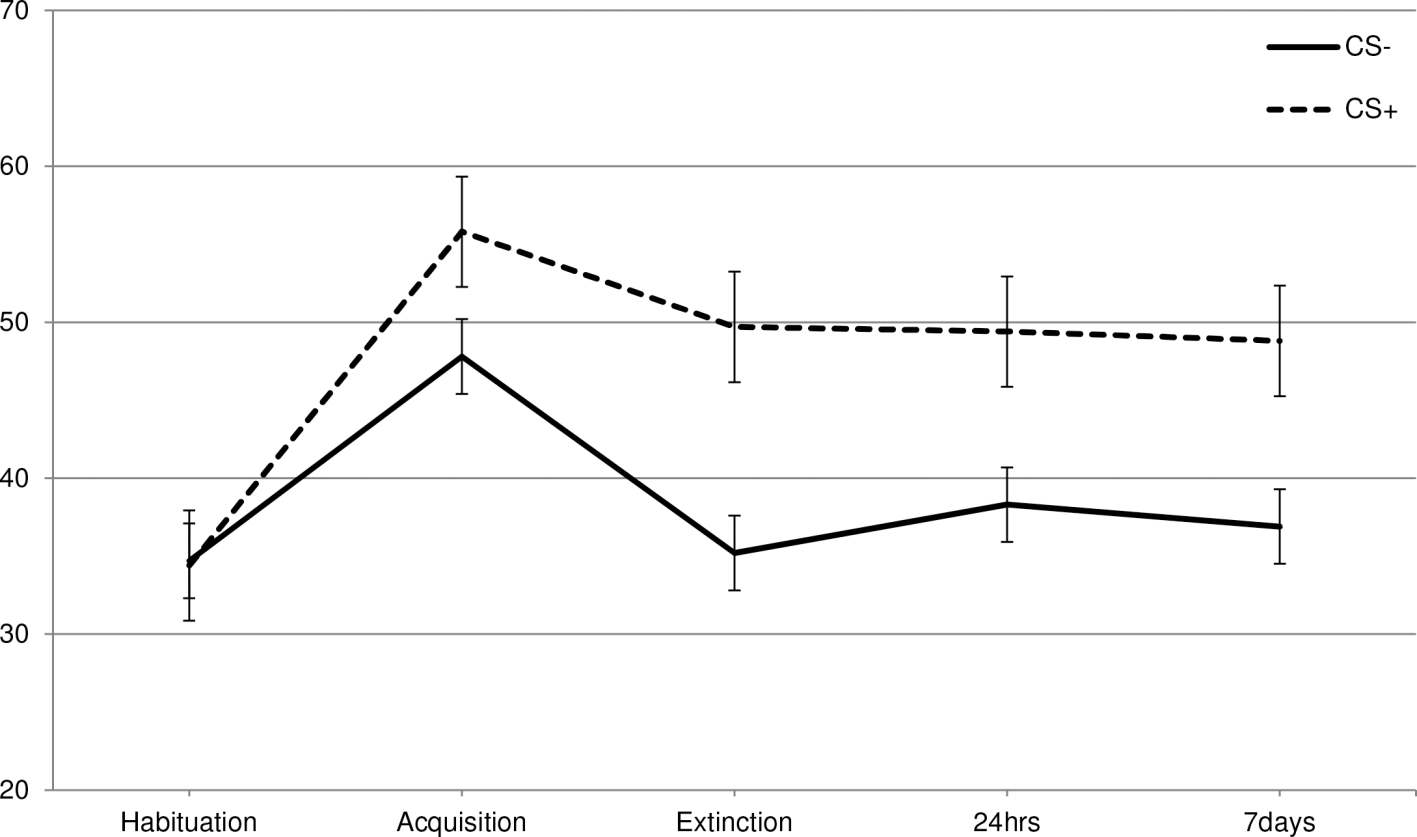
Self-reported disgust. Total scores in the different phases (habituation, acquisition, extinction, 24hrs and 7 days) of the self-reported disgust.

**Fig 2:**
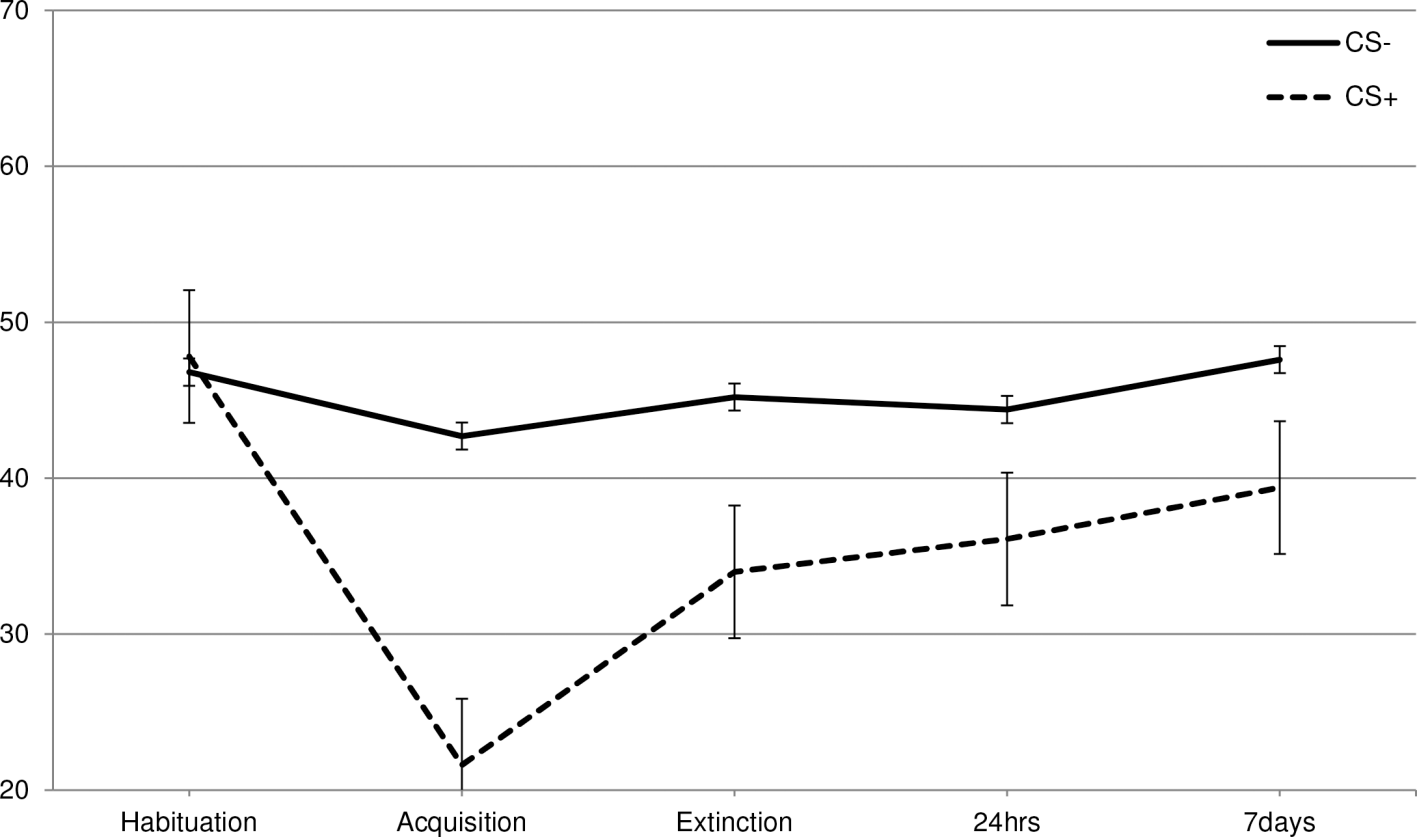
Self-reported willingness-to-eat. Total scores in the different phases (habituation, acquisition, extinction, 24hrs and 7 days) of the self-reported willingness-to-eat.

**Fig 3:**
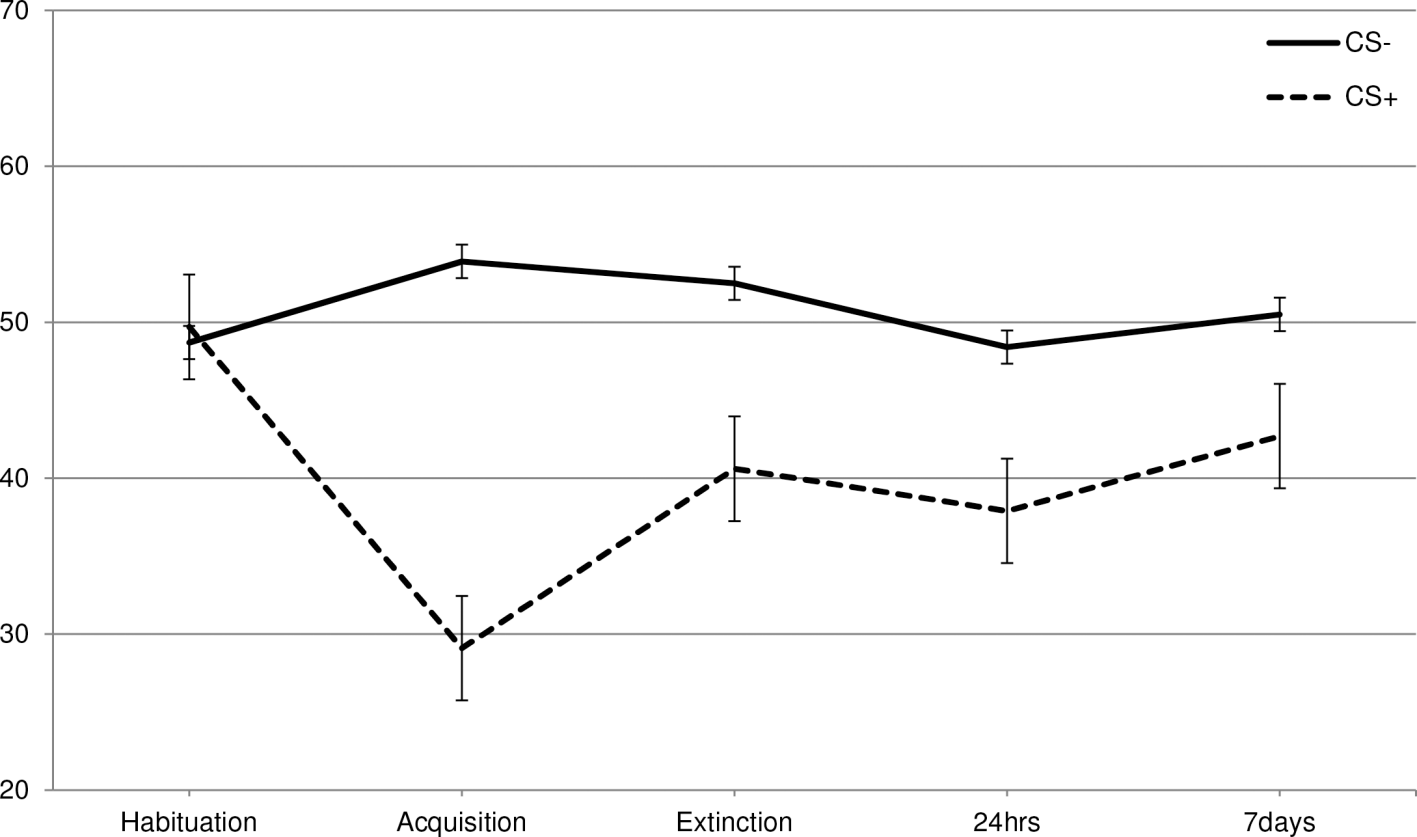
Self-reported valence. Total scores in the different phases (habituation, acquisition, extinction, 24hrs and 7 days) of the self-reported valence.

To test if disgust could indeed be learned via classical conditioning, each of the three VASs (disgust, willingness-to-eat, and valence) were subjected to a 2 CS (CS+, CS-) * 2 Phase (habituation, acquisition) RM-ANOVA. Disgust increased in response to the CS+ following acquisition as was indicated by a CS * Phase interaction [F (1, 265) = 7.5, p = .007, $\eta_{p}^{2}$ = .027] (S1 Table, Figs 1–3). In line with the disgust ratings, participants indicated that following acquisition they were less willing to eat the CS+ as indicated by the CS * Phase interaction [F (1, 265) = 126.5, p < .001, $\eta_{p}^{2}$ = .323]. A similar pattern was also observed for the valence ratings [F (1, 265) = 189.4, p < .001, $\eta_{p}^{2}$ = .417].

Additional paired comparisons showed that in habituation there were no significant differences between the CS+ and CS- [disgust: *t* (265) = 0.125, p = .901; willingness-to-eat: *t* (265) = 0.518, p = .605; valence: *t* (265) = 0.449, p = .654]. Following acquisition, the differences between the CS+ and CS- were significant for all three VAS-dimensions [disgust: *t* (265) = 3.1, p = .002; willingness-to-eat: *t* (265) = 11.4, p < .001; valence: *t* (265) = 12.3, p < .001].

#### Was disgust (also) learned as evidenced by differential EMG activity?

**Fig 4:**
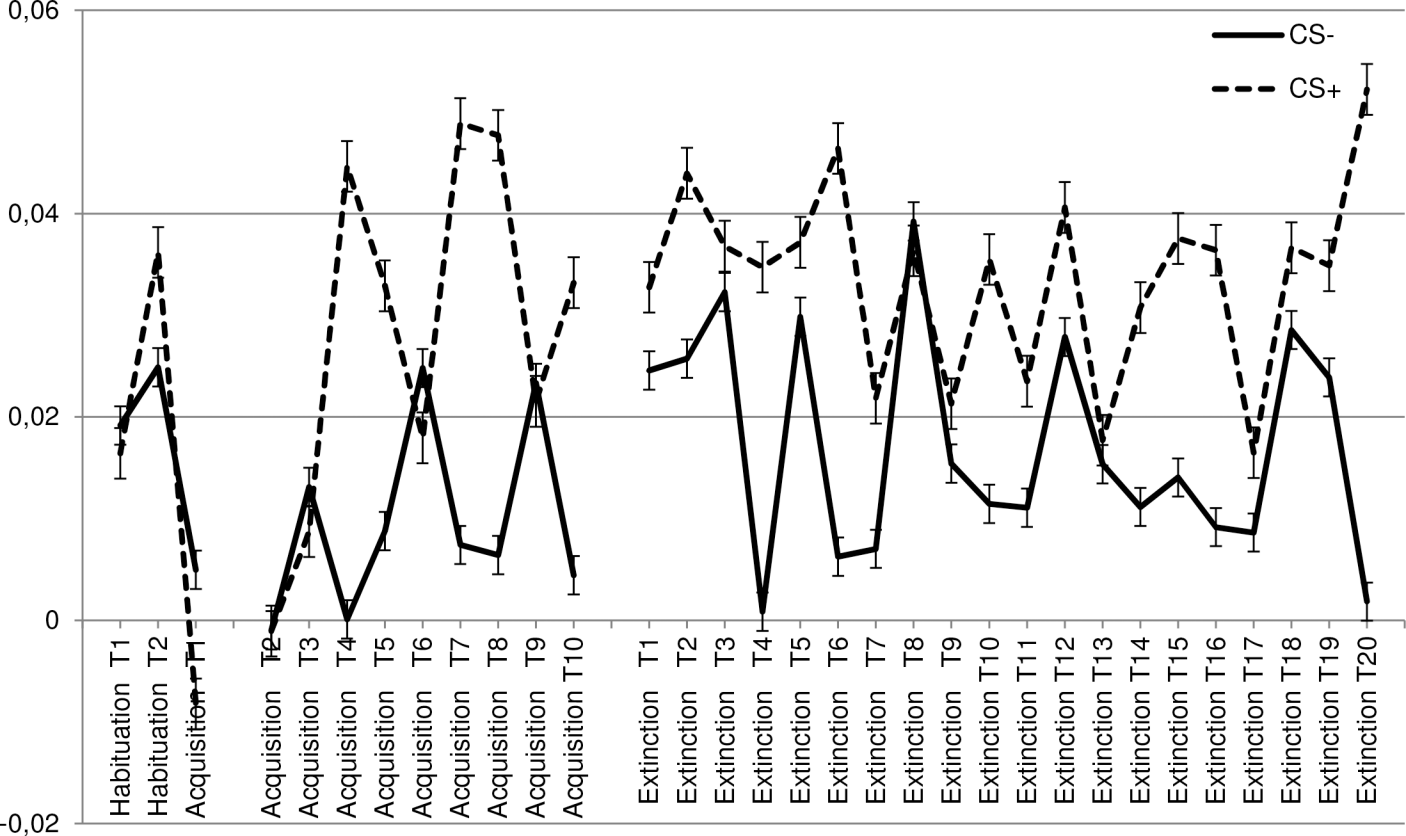
Corrugator conditioned response. Mean response in the different phases (habituation, acquisition, extinction) of the facial corrugator towards the CS+ and CS−.

**Fig 5:**
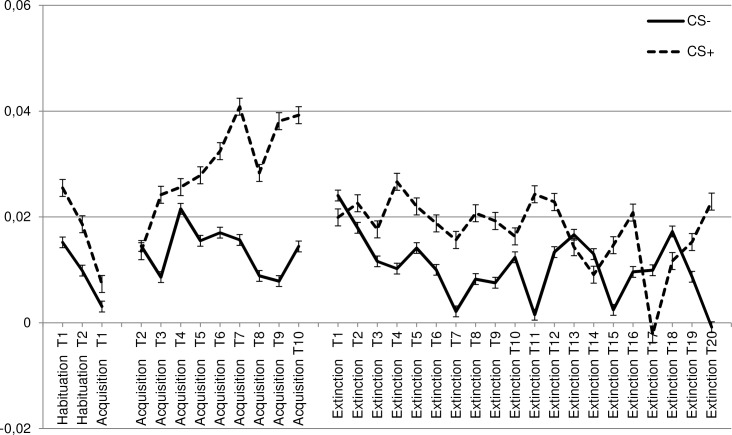
Levator conditioned response. Mean response in the different phases (habituation, acquisition, extinction) of the facial levator towards the CS+ and CS−.

Data were subjected to a 2 CS (CS+, CS-) * 2 Phase (habituation, acquisition) * 2 Muscle (corrugator, levator) RM-ANOVA. Important in the context of the present study was that the CS * Phase interaction was significant [F (1, 130) = 5.4, p = .022, $\eta_{p}^{2}$ = .040], indicating that conditioning resulted in relatively enhanced EMG responsivity towards the CS+ vs. relatively reduced EMG responsivity towards the CS- (S2 Table, Figs 4 and 5). This effect was similar for both muscles, as evidenced by the absence of a significant CS * Phase * Muscle interaction [F (1, 130) = 1.5, p = .225, $\eta_{p}^{2}$ = .011].

### Extinguishing disgust

#### Effect of experimental condition on the extinction of conditioned subjective disgust

To test the influence of condition on extinction, each of the three VASs (disgust, willingness-to-eat, and valence) were subjected to 2 CS (CS+, CS-) * 2 Phase (acquisition, extinction) * 4 Condition (no-exposure, BAT, active enforcer, inactive enforcer) RM-ANOVA.

For disgust there was a significant CS * Phase interaction [F (1, 262) = 5.2, p = .024, $\eta_{p}^{2}$ = .02], showing that overall conditioned disgust was sensitive to extinction (S1 Table, Fig 1). The effect of extinction was similar for all conditions, as evidenced by the absence of a significant CS * Phase * Condition interaction [F (1, 262) = 1.6, p = .196, $\eta_{p}^{2}$ = .02]. Although the difference in disgust responding between the CS+ and CS- overall declined following extinction, the post-extinction difference remained substantial and statistically significant, [*t* (265) = 6.9, p < .001].

For willingness-to-eat there was also a significant CS * Phase interaction [F (1, 262) = 55.8, p < .001, $\eta_{p}^{2}$ = .175], indicating that overall the reported willingness-to-eat increased stronger for the CS+ than for the CS- (S1 Table, Fig 2). Importantly, the strength of extinction varied across conditions as evidenced by a significant three-way interaction CS * Phase * Condition [F (3, 262) = 10.0, p < .001, $\eta_{p}^{2}$ = .103]. A series of RM-ANOVAs were conducted with 2 CS (CS+, CS-) * Phase (acquisition, extinction) * 2 Condition (no-exposure vs. BAT or active enforcer or inactive enforcer) to investigate which type of exposure was most effective in increasing willingness-to-eat compared to no-exposure. A Bonferroni correction was applied (0.05/3 = 0.0167). For the analysis contrasting the BAT-condition with the no-exposure condition there was a significant three-way CS * Phase * Condition interaction [F (1, 134) = 24.6, p < .001, $\eta_{p}^{2}$ = .155]. Subsequent t-tests indicated that following acquisition there were no differences between the conditions in the rating of the CS- [*t* (134) = 0.354, p = .72, M_Dif_ = 1.9, SE_Dif_ = 5.5] and the CS+ [*t* (134) = 0.608, p = .54, M_Dif_ = 2.1, SE_Dif_ = 3.4]. However, following extinction the ratings for the CS+ differed between conditions [*t* (134) = 4.1, p < .001, M_Dif_ = 18.5, SE_Dif_ = 4.6]. Participants were more willing to eat the CS+ following extinction when the CS-only exposure was preceded by a BAT. A similar difference between conditions was absent for the CS- [*t* (134) = 0.04, p = .97, M_Dif_ = 0.20, SE_Dif_ = 5.5].

For the analysis contrasting the active enforcer with the no-exposure control condition, the CS * Phase * Condition interaction did not reach significance [F (1, 134) = 2.5, p = .119, $\eta_{p}^{2}$ = .018], indicating that there was no reliable difference between the active enforcer and no-exposure condition with respect to their impact on extinction and ability to increase willingness-to-eat the CS+.

Also for the analysis contrasting the inactive enforcer with the no-exposure condition, there was no significant three-way interaction [F (1, 132) = 0.08, p = .799, $\eta_{p}^{2}$ = .001], indicating no differential impact on willingness-to-eat by the inactive enforcer and no-exposure condition.

For valence there was a significant CS * Phase interaction [F (1, 262) = 90.1, p < .001, $\eta_{p}^{2}$ = .256], indicating that overall the liking of the CS+ again increased following extinction (S1 Table, Fig 3). This effect varied across conditions as was evidenced by a significant CS * Phase * Condition interaction [F (3, 262) = 2.7, p = .045, $\eta_{p}^{2}$ = .030]. To investigate which type of exposure was more effective in increasing the liking for the CS+ than no-exposure, a RM-ANOVA was conducted with 2 CS (CS+, CS-) * 2 Phase (acquisition, extinction) * 2 Condition (no-exposure vs. BAT or active enforcer or inactive enforcer). A Bonferroni correction was applied to encounter for multiple comparisons (0.05/3 = 0.0167). For the analysis contrasting the BAT condition with the control condition, a significant CS * Phase * Condition interaction was observed [F (1, 134) = 7.7, p = .006, $\eta_{p}^{2}$ = .054]. Subsequent t-tests showed that following acquisition there were no differences between both conditions for neither the CS- [*t* (134) = 0.46, p = .65, M_Dif_ = 1.94, SE_Dif_ = 4.2] nor the CS+ [*t* (134) = 0.922, p = .36, M_Dif_ = 3.66, SE_Dif_ = 4.0]. Following extinction there were no differences between the conditions for the CS- [*t* (134) = 0.723, p = .47, M_Dif_ = 3.0, SE_Dif_ = 4.2], while the CS+ was liked significantly more in the BAT condition compared to the no-exposure condition [*t* (134) = 3.5, p = .001, M_Dif_ = 14.6, SE_Dif_ = 4.2] (S1 Table, Fig 3).

For the analysis of the active enforcer vs. control condition the CS * Phase * Condition interaction did not approach significance [F (1, 134) = 0.078, p = .78, $\eta_{p}^{2}$ = .001]. Thus the active enforcer condition was not more effective in increasing the liking of the CS+ than the no-exposure condition.

Also for the analysis contrasting the inactive enforcer condition with the no-exposure control condition there was no significant CS * Phase * Condition interaction [F (1, 132) = .37, p = .55, $\eta_{p}^{2}$ = .003]. Thus the inactive exposure condition was not more effective in increasing the liking of the CS+ than the mere CS-only no-exposure condition.

#### Facial EMG

To test the impact of condition on disgust unlearning at the level of facial EMG, data were subjected to a 2 CS (CS+, CS-) * 2 Phase (acquisition, extinction) * 2 Muscle (corrugator, levator) * 4 Condition (no-exposure, BAT, active enforcer, inactive enforcer) RM-ANOVA with Condition as a between-subject variable. Most important for the present context, there was no significant CS * Phase interaction [F (1, 127) = 1.3, p = .26, $\eta_{p}^{2}$ = .010], and neither a significant CS * Phase * Muscle interaction [F (1, 127) = .531, p = .467, $\eta_{p}^{2}$ = .004], or a significant CS * Phase * Muscle * Condition interaction [F (1, 127) = 1.1, p = .354, $\eta_{p}^{2}$ = .025]. Thus no evidence emerged to indicate that there was a stronger influence of the extinction procedures on the responsivity of CS+ than on the responsivity of the CS-. However, there was a significant main effect of CS [F (1, 127) = 16.4, p < .001, $\eta_{p}^{2}$ = .114], indicating that the CS+ generally elicited stronger EMG activity than the CS- (S2 Table, Figs 4 and 5). Thus it seemed that the conditioned EMG response was unaffected by the extinction procedures. Unrelated to the aim of the study there was also a significant Phase * Muscle * Condition interaction [F (3, 127) = 2.8, p = .045, $\eta_{p}^{2}$ = .061].

### Approach behaviour

A *X*^*2*^ test was conducted to test whether percentage of participants eating the CS+/CS- varied between conditions. The test provided no evidence that the percentage of participants eating the CS+ and CS- was affected by condition [*X*^*2*^ (3 (N = 132)) = 0.74, p = .86].

### Long(er) term effect of experimental condition on subjective indices of disgust

To test the long-term effects of the extinction procedure as a function of condition, each of the three VASs (disgust, willingness-to-eat, and valence) were subjected to 2 CS (CS+, CS-) * 3 Phase (extinction, 24hours, 7 days) * 4 Condition (no-exposure, BAT, active enforcer, inactive enforcer) RM-ANOVA.

For disgust there was a significant three-way interaction CS * Phase * Condition [F (6, 524) = 4.9, p < .001, $\eta_{p}^{2}$ = .053] (S1 Table, Fig 1). To investigate which exposure condition deviated from the no-exposure condition, a RM-ANOVA was conducted with 2 CS (CS+, CS-) * 3 Phase (extinction, 24hours, 7 days) * 2 Condition (no-exposure vs. BAT or active enforcer or inactive enforcer). A Bonferroni correction was applied to encounter for multiple comparisons (0.05/3 = 0.0167).

For the analysis contrasting the BAT condition with the no-exposure condition there was a significant CS * Phase * Condition interaction [F (2, 133) = 5.0, p = .008, $\eta_{p}^{2}$ = .07]. Subsequent t-tests showed that immediately after extinction there were no significant differences between both conditions regarding the disgust ratings of the CS- [*t* (134) = .18, p .857, M_Dif_ = 0.806, SE_Dif_ = 4.5] and the CS+ [*t* (134) = 1.9, p = .056, M_Dif_ = 9.7, SE_Dif_ = 5.1). There were also no significant differences between the conditions regarding the disgust ratings after 24 hours [CS-: *t* (134) = 957, p = .34, M_Dif_ = 4.1, SE_Dif_ = 4.3; CS+: *t* (134) = 0.07, p = .942, M_Dif_ = 0.38, SE_Dif_ = 5.3]. After seven days, however, differences emerged between the conditions: In the BAT condition the CS+ was rated significantly less disgusting than in the no-exposure condition [*t* (134) = 2.0, p = .04, M_Dif_ = 9.2, SE_Dif_ = 4.5], while there was no difference between both conditions for the disgust rating of the CS- [*t* (134) = 0.44, p = .66, M_Dif_ = 1.8, SE_Dif_ = 4.1].

There was also a significant three-way interaction for the active enforcer condition [F (2, 133) = 4.4, p = .015, $\eta_{p}^{2}$ = .062]. Subsequent t-tests showed that immediately after extinction there were no differences between the groups regarding their subjective disgust ratings [CS-: *t* (134) = 0.59, p = 0.56, M_Dif_ = 2.6, SE_Dif_ = 4.5; CS+: *t* (134) = 1.6, p = .12, M_Dif_ = 8.2, SE_Dif_ = 5.2]. After 24 hours, there were no differences between the conditions for the CS- [*t* (134) = 0.61, p = .54, M_Dif_ = 2.5, SE_Dif_ = 4.1], but in the active enforcer condition the CS+ was rated significantly less disgusting than the no-exposure condition [*t* (134) = 2.1, p = .034, M_Dif_ = 10.90, SE_Dif_ = 5.1]. This pattern was still present seven days after conditioning [CS-: *t* (134) = 0.93, p = 0.35, M_Dif_ = 3.5, SE_Dif_ = 3.8; CS+: *t* (134) = 2.6, p = .012, M_Dif_ = 12.7, SE_Dif_ = 5.0].

For the inactive enforcer condition the three-way interaction did not reach the Bonferroni-corrected level of significance [F (2, 131) = 3.1, p = .049, $\eta_{p}^{2}$ = .045]. Thus there was no evidence to indicate that the inactive enforcer condition differentially affected the long-term course of subjective disgust.

For willingness-to-eat a three-way interaction CS * Phase * Condition was observed [F (6, 524) = 2.4, p = .03, $\eta_{p}^{2}$ = .027] (S1 Table, Fig 2). To further investigate this effect, three 2 CS (CS+, CS-) * 3 Phase (extinction, 24hours, 7days) * 2 Condition (no-exposure vs. BAT or active enforcer or inactive enforcer) RM-ANOVA were conducted and interactions were interpreted with a Bonferroni corrected level of alpha (0.05/3 = 0.0167). When comparing the no-exposure with the BAT condition a significant CS * Phase * Condition interaction was present [F (2, 133) = 6.3, p = .002, $\eta_{p}^{2}$ = .087]. As already reported above, subsequent t-tests showed that immediately following the extinction procedure there was a significant difference between conditions regarding the CS+[*t* (134) = 4.1, p = < .001, M_Dif_ = 18.5, SE_Dif_ = 4.6] but not for the CS- [*t* (134) = 0.04, p = .97, M_Dif_ = 0.20, SE_Dif_ = 5.5]. This difference between conditions regarding the CS+ was no longer significant after 24 hours [*t* (134) = 1.6, p = .10, M_Dif_ = 7.6, SE_Dif_ = 4.6]. After seven days, however, the BAT condition again showed a significantly higher willingness-to-eat the CS+ ratings than the no-exposure condition [*t* (134) = 2.2, p = .03, M_Dif_ = 9.5, SE_Dif_ = 4.3]. A similar difference was absent for the CS- [*t* (134) = 0.16, p = .55, M_Dif_ = 2.7, SE_Dif_ = 4.4].

For the analysis contrasting the active enforcer condition with the no-exposure condition, the CS * Phase * Condition interaction was not significant [F (2, 133) = 2.7, p .071, $\eta_{p}^{2}$ = .039]. Thus there was no evidence to indicate a differential impact of the active enforcer condition on the longer term course of the willingness-to-eat the CS+.

Also for the analysis contrasting the inactive enforcer condition with the no-exposure condition, the CS * Phase * Condition interaction did not reach the Bonferroni-corrected level of significance [F (2, 131) = 3.6, p = .029, $\eta_{p}^{2}$ = .053].

For valence, in line with expectations, the CS * Phase interaction reached significance [F (3, 262) = 3.90, p = .021, $\eta_{p}^{2}$ = .015]. As can be seen in S1 Table and in Fig 3, this finding indicated that the differential appreciation of the CS+ versus the CS- reduced over time. This pattern appeared independent of experimental condition as was evidenced by the absence of a significant CS * Phase * Condition interaction [F (6, 524) = 0.64, p = .697, $\eta_{p}^{2}$ = .007].

## Discussion

In this study we tested the efficacy of providing additional safety information vs. traditional CS-only exposure in the weakening of conditioned disgust. The main findings can be summarized as follows: i) Consolidating our previous findings, the current differential conditioning procedure was successful in enhancing the disgust response towards the CS+ as indexed by self-report indices and facial EMG; ii) Although the subjective disgust response remained substantial following the extinction procedure, the subjective indices of conditioned disgust overall declined. In contrast, the enhanced EMG-responding towards the CS+ remained unaffected by the extinction procedures. iii) Moreover, adding a Behavioural Approach Task (BAT) to the CS-only extinction procedure promoted the immediate post-extinction increase in willingness-to-eat and the liking of the CS+. A similar immediate effect was absent for the active enforcer and inactive enforcer conditions. iv). Finally, the beneficial effect of adding a BAT on the extinction of subjective disgust was still evident at one-week follow up. The active enforcer condition showed a similar long-term benefit.

### Disgust learning

Corroborating previous work, the current conditioning procedure successfully enhanced disgust towards the CS+ as indexed by self-reported disgust (small to medium effect size), and willingness-to-eat (large effect size). In addition, the paired CS-US presentations resulted in a general decrease of the liking of the CS+ (large effect size), which can be interpreted as an evaluative conditioning effect. Consistent with this pattern of self-reported findings, the conditioning procedure also gave rise to a stronger response towards the CS+ than the CS- as indexed by facial EMG of the levator and corrugator muscles (small to medium effect size). The enhanced activity of the corrugator muscle may reflect a more generally negative appreciation of the CS+ following acquisition that was also found in previous research [18], whereas the enhanced activity of the (facial) levator muscle suggests that the current conditioning procedure was also effective in eliciting more disgust-specific facial EMG responding. The overall pattern of findings matches those of conceptually similar work using behavioural measures to index disgust-conditioning [16–17, 36]. Thus, this part of the study lends further support to the view that classical conditioning is an effective procedure as a pathway for learning disgust.

### Strategies to unlearn disgust

Previous research, both experimental lab designs and clinical treatment studies have consistently showed that disgust is relatively resistant to extinction [37]. Such perseverant maladaptive disgust responses disrupt optimal treatment outcomes and might play a role in relapse for various (disgust-driven) psychopathologies [37]. In line with the view that disgust-learning can be considered as a sticky form of learning [15], the current study failed to find an immediate effect of extinction procedures on conditioned disgust responding as indexed by facial EMG. In addition, also the increased self-reported disgust and lowered willingness-to-eat the CS+ remained substantial following the extinction procedures.

The current study was primarily designed to test whether additional safety knowledge would help to reduce disgust via challenging the acquired threat value attached to the CS+. Previous research provided preliminary evidence that handling/smelling/eating/ the CS+ in the context of a BAT promoted the efficacy of CS-only exposure as a procedure to reduce subjective disgust [18]. Thus the first aim of this study was to test the robustness of this earlier finding while controlling for the prolonged exposure time that was implied by adding a BAT before the CS-only extinction procedure. In other words, the current design allowed us to rule out that a beneficial effect of adding a BAT to extinction would be merely driven by prolonged exposure time.

The current findings showed that indeed this earlier finding could be considered as a reliable phenomenon, in that immediately post-extinction the BAT condition was [the most] effective in increasing both the willingness-to-eat (large effect size) and the liking of the CS+ (median effect size). In contrast, the active enforcer, and the inactive enforcer condition showed small effects that were not significant. Thus the immediate beneficial effects were absent for the active and the inactive enforcer conditions. Probably this is due to the fact that in the active enforcer condition the participants were only visually exposed to an actress eating the food items representing the CSs in the role of an observant, whereas in the BAT condition participants received more information at first-hand to counteract the initial threat value attached to the CS+. Moreover in the BAT condition, participants had the opportunity to more actively explore the CS+ than in the other three conditions. Supporting the relevance of adding more convincing safety information rather than mere exposure or by simply providing strategies in which the participant is an observer, is the notable finding that the beneficial effects were still evident at one-week follow up (small to medium effect size).

It should be acknowledged, however, that the immediate beneficial effect was restricted to self-report indices. No evidence emerged to indicate that this procedure/additional information also influenced actual eating behaviour. Also, the conditioned disgust expression as indexed by facial EMG appeared insensitive to the safety information provided via BAT. Yet, the absence of a beneficial effect on the actual eating could be due to demand [38]. Asking participants to have a bite from the food items at the university lab, might be quite different from when the same offer is done without external pressure, and/or out of the safety-lab-context. Besides, different mechanisms may moderate the self-reported appraisal of a stimulus as opposed to the actual behaviour. On the other hand, the absence of extinction as indexed by the physiological facial response may indicate that to counteract the acquired reflexive responses, one may need to employ stronger strategies and more convincing information in order to effectively neutralise the (disgust-relevant) more automatically activated threat value of the CS+ [23]. As a final limitation it should be mentioned that we asked participants to rate their hunger (How hungry are you at this moment) to control for potential differences in hunger across conditions instead of their pre-experimental food intake, whereas the latter might be a better predictor of actual eating behaviour. Thus although conditions did not differ with regard to participants’ hunger ratings, it cannot be ruled out that there might still have been differences in caloric intake prior to the experiment between conditions.

The findings of this study may also help understand why traditional exposure treatment is typically not very efficient in reducing disgust [14, 37, 39]. More specifically, the current findings suggest that effective exposure exercises should provide individuals with a compelling experience that it is perfectly safe to have intense physical contact with the disgust eliciting stimulus. Mere visual exposure, or exposure without prolonged physical contact is probably insufficient to effectively convey the information that a particular stimulus is free from invisible yet threatening contaminants. The concrete type of exercise that is necessary to achieve this safe experience will of course depend on the type of disgust elicitor that is involved, and may thus also vary across disgust-relevant disorders (e.g., spider phobia vs. OCD). Further, the current findings may also help explain why previous attempts to use counterconditioning procedures to reduce disgust were largely ineffective [5]. Although counterconditioning may be helpful to reduce the negative valence of the disgusting stimulus per se, it does not challenge the signal value of the CS. Thus even after a successful counterconditioning intervention, the CS may still be experienced as a cue for the presence of pathogens.

In conclusion, the current findings replicated previous research indicating that disgust can be learned via classical conditioning procedures. Most important, the findings of this study corroborated the view that the mere visual presentation of CS-only is not an efficient means to reduce acquired disgust because it may not provide the type of safety information required/necessary to undermine the threat-value of invisible threats, and thereby to remove its properties to elicit a disgust response. Given the difficulty to eliminate the maladaptive disgust responding in various psychopathologies, this finding might inspire future research to develop and test new strategies that are effective in eliminating persistent maladaptive disgust.

## Supporting Information

S1 Table VAS scores per Phase per CS Type. Subjective evaluation for each of the 5-phases (i.e., habituation, acquisition, extinction, 24hrs, 7 days) on the 3-dimensions (i.e., disgust, willingness-to-eat, and valence) as measured on the VAS per condition (1, no exposure, 2, BAT, 3, active enforcer, 4, inactive enforcer). Different letters in superscript indicate significant interactions based on RM-ANOVA: CS*Phase interaction disgust learning, a,b: p < .001; c,d: p = .007; CS*Phase interaction disgust unlearning, e,f: p < .001, g,h: p = .024; CS*Phase*Condition disgust unlearning: i,j: p < .001; k,l: p = .045; CS*Phase*Condition long-term: m,n,o: p < .001; p,q,r: p = .03; CS*Phase*Condition disgust unlearning (no exposure vs. BAT): s,t p ≤ .006; CS*Phase*Condition long term (no exposure vs. BAT), u,v,w: p ≤ .008; CS*Phase*Condition long term (no exposure vs. active enforcer), x,y,z: p = .015. Different numbers in superscript indicate significant differences based on t-test: CS- vs. CS+ within phase, 1,2: p < .001; 3,4 p = .002; difference CS+ across conditions no-exposure vs. BAT: 5,6 p ≤ .008; difference CS+ across conditions no-exposure vs. active enforcer: 7,8 p ≤ .034.
(DOCX)

S2 Table Muscular response per Phase per CS. Mean EMG activity in microvolt for muscle type (corrugator, levator) as a function of phase (habituation, acquisition, extinction) and type of CS per group.
(DOCX)

S1 Appendix Manipulation checks for the conditioned stimuli (CS). (DOCX)
