# Supplementary material for: Optimising Extinction of Conditioned Disgust
Source: PLoS One. 2016 Feb 5;11(2):e0148626. doi: 10.1371/journal.pone.0148626 (PMC4743916; doi:10.1371/journal.pone.0148626)
Supplement: S1 Table — Subjective evaluation for each of the 5-phases (i.e., habituation, acquisition, extinction, 24hrs, 7 days) on the 3-dimensions (i.e., disgust, willingness-to-eat, and valence) as measured on the VAS per condition (1, no exposure, 2, BAT, 3, active enforcer, 4, inactive enforcer). Different letters in superscript indicate significant interactions based on RM-ANOVA: CS*Phase interaction disgust learning, a,b: p < .001; c,d: p = .007; CS*Phase interaction disgust unlearning, e,f: p < .001, g,h: p = .024; CS*Phase*Condition disgust unlearning: i,j: p < .001; k,l: p = .045; CS*Phase*Condition long-term: m,n,o: p < .001; p,q,r: p = .03; CS*Phase*Condition disgust unlearning (no exposure vs. BAT): s,t p ≤ .006; CS*Phase*Condition long term (no exposure vs. BAT), u,v,w: p ≤ .008; CS*Phase*Condition long term (no exposure vs. active enforcer), x,y,z: p = .015. Different numbers in superscript indicate significant differences based on t-test: CS- vs. CS+ within phase, 1,2: p < .001; 3,4 p = .002; difference CS+ across conditions no-exposure vs. BAT: 5,6 p ≤ .008; difference CS+ across conditions no-exposure vs. active enforcer: 7,8 p ≤ .034. (DOCX) [file pone.0148626.s001.docx]

|  | | Habituation | | Acquisition | | Extinction | | 24hrs | | 7days | |
| --- | --- | --- | --- | --- | --- | --- | --- | --- | --- | --- | --- |
|  | | CS- | CS+ | CS- | CS+ | CS- | CS+ | CS- | CS+ | CS- | CS+ |
| VAS | Condition | M (SD) | M (SD) | M  (SD) | M  (SD) | M  (SD) | M  (SD) | M  (SD) | M  (SD) | M  (SD) | M  (SD) |
| Disgust | No exposure | 33.8 (25.8) | 40 (29.6) | 47.6 (29.5) | 58.2 (33.9) | 31.2 (25.6)^u,x^ | 54.3 (29.8)^u,x^ | 37.4 (21.8)^v,y^ | 50.9 (30.2)^v,y,7^ | 35 (20.3)^w,z^ | 52.7 (25.9)^w,z,5,7^ |
|  | BAT | 31.2 (23.3) | 29.9 (29.5) | 49.2 (33.3) | 51.3 (32.4) | 30.4 (26.6)^u^ | 44.6 (29.1)^u^ | 33.3 (27.9)^v^ | 50.5 (31.5)^v^ | 33.2 (27.2)^w^ | 43.5 (26.9)^w,6^ |
|  | Inactive enforcer | 35.7 (24.7) | 29.9 (26.1) | 41.8 (29.1) | 51.4  (28) | 33.8 (26.4) | 46.2 (31.3) | 35 (25.4) | 40 (28.7) | 31.5 (23.9) | 40.1 (31.8) |
|  | Active enforcer | 38.4 (31.4) | 39.3 (27.6) | 52.6 (33.5) | 62.1  (31) | 45.8 (30.8)^x^ | 53.5 (30.8)^x^ | 47.7 (30.7)^y^ | 56.4 (29.4)^y,8^ | 48.5 (29.3)^z^ | 58.9 (25.6)^z,8^ |
|  | Total | 34.7 (26.4)^c^ | 34.4 (28.6)^c^ | 47.8 (31.4)^d,g,3^ | 55.8 (31.6)^d,g,4^ | 35.2 (27.9)^h,m,1^ | 49.7 (30.4)^h,m,2^ | 38.3 (27.0)^n,1^ | 49.4 (30.4)^n,2^ | 36.9 (26.0)^o,1^ | 48.8 (28.4)^o,2^ |
| Willingness to eat | No exposure | 47.5 (24.7) | 44.3 (25.4) | 45.8 (31.8)^s^ | 16.6 (19.3)^s^ | 49.3 (31.2)^t,u^ | 23.9 (21.8)^t,u,5^ | 45.3 (24.6)^v^ | 30.5 (23.3)^v^ | 49.1 (23.7)^w^ | 35.3 (23.3)^w,5^ |
|  | BAT | 46.0 (30.2) | 49.0 (30.6) | 47.8 (32.8)^s^ | 18.7 (20.4)^s^ | 49.5 (32.4)^t,u^ | 42.4 (30.3)^t,u,6^ | 47.4 (29.2)^v^ | 38.1 (30.2)^v^ | 51.7 (27.5)^w^ | 44.8 (27.1)^w,6^ |
|  | Inactive enforcer | 47.6 (24.7) | 54.2 (28.6) | 41.3 (25.4) | 28.6 (26.3) | 44.4 (27.4) | 40.8 (31.0) | 43.7 (27.7) | 44.3 (30.3) | 48.1 (29.1) | 43.1 (30.1) |
|  | Active enforcer | 45.9 (29.4) | 43.9 (26.2) | 35.5 (31.1) | 23.0 (29.0) | 37.1 (28.7) | 29.3 (29.3) | 40.8 (28.9) | 31.7 (26.2) | 41.3 (26.6) | 34.3 (25.3) |
|  | Total | 46.8 (27.2)^a^ | 47.8 (27.9)^a^ | 42.7 (30.6)^b,e,i,1^ | 21.6 (24.3)^b,e,i,2^ | 45.2 (30.3)^f,j,p,1^ | 34.0 (29.2)^f,j,p,2^ | 44.4 (27.6)^q,1^ | 36.1 (28.0)^q,2^ | 47.6 (26.9)^r,1^ | 39.4 (26.8)^r,2^ |
| Valence | No exposure | 48.2 (21.8) | 45.9 (20.9) | 56.7 (22.6)^s^ | 25.1 (21.0)^s^ | 55.1 (23.8)^t^ | 33.1 (21.1)^t,5^ | 48.8 (21.8) | 30.6 (19.8) | 51.4 (19.3) | 37.7 (21.3) |
|  | BAT | 52.8 (22.4) | 52.6 (27.6) | 58.7 (26.4)^s^ | 28.8 (25.2)^s^ | 58.1 (24.9)^t^ | 47.7 (27.4)^t,6^ | 52.2 (26.0) | 41.0 (28.8) | 55.7 (23.9) | 48.8 (26.2) |
|  | Inactive enforcer | 48.4 (20.6) | 54.5 (26.8) | 51.7 (21.0) | 37.2 (27.1) | 51.5 (23.5) | 47.5 (26.5) | 48.7 (23.9) | 46.8 (26.2) | 50.1 (24.6) | 48.2 (29.7) |
|  | Active enforcer | 45.6 (27.3) | 45.8 (23.6) | 48.1 (28.2) | 25.4 (25.1) | 45.1 (24.9) | 34.3 (26.7) | 43.9 (27.0) | 33.8 (26.7) | 44.5 (24.9) | 36.1 (25.3) |
|  | Total | 48.7 (23.1)^a^ | 49.7 (25.0)^a^ | 53.9 (24.9)^b,e,k,1^ | 29.1 (25.0)^b,e,k,2^ | 52.5 (24.6)^f,l,1^ | 40.6 (26.3)^f,l,2^ | 48.4 (24.7)^1^ | 37.9 (26.1)^2^ | 50.5 (23.4)^1^ | 42.7 (26.2)^2^ |

Table S1: Title S1. VAS scores per Phase per CS Type

Table S1: Legend S1. Subjective evaluation for each of the 5-phases (i.e., habituation, acquisition, extinction, 24hrs, 7 days) on the 3-dimensions (i.e., disgust, willingness-to-eat, and valence) as measured on the VAS per condition (1, no exposure, 2, BAT, 3, active enforcer, 4, inactive enforcer). Different letters in superscript indicate significant interactions based on RM-ANOVA:

CS*Phase interaction disgust learning, a,b: p < .001; c,d: p = .007; CS*Phase interaction disgust unlearning, e,f: p < .001, g,h: p=.024; CS*Phase*Condition disgust unlearning: i,j: p < .001; k,l: p = .045; CS*Phase*Condition long-term: m,n,o: p <.001; p,q,r: p = .03; CS*Phase*Condition disgust unlearning (no exposure vs. BAT): s,t p ≤ .006; CS*Phase*Condition long term (no exposure vs. BAT), u,v,w: p ≤ .008; CS*Phase*Condition long term (no exposure vs. active enforcer), x,y,z: p = .015.

Different numbers in superscript indicate significant differences based on t-test:

CS- vs. CS+ within phase, 1,2: p < .001; 3,4 p = .002; difference CS+ across conditions no-exposure vs. BAT: 5,6 p ≤ .008; difference CS+ across conditions no-exposure vs. active enforcer: 7,8 p ≤ .034.
