# Supplementary material for: Optimising Extinction of Conditioned Disgust
Source: PLoS One. 2016 Feb 5;11(2):e0148626. doi: 10.1371/journal.pone.0148626 (PMC4743916; doi:10.1371/journal.pone.0148626)
Supplement: S2 Table — Mean EMG activity in microvolt for muscle type (corrugator, levator) as a function of phase (habituation, acquisition, extinction) and type of CS per group. (DOCX) [file pone.0148626.s002.docx]

|  | | | | | | | |
| --- | --- | --- | --- | --- | --- | --- | --- |
|  | | Habituation | | Acquisition | | Extinction | |
|  | | CS- | CS+ | CS- | CS+ | CS- | CS+ |
| Muscle | Condition | M (SD) | M (SD) | M (SD) | M (SD) | M (SD) | M (SD) |
| Corrugator | No exposure | 0.010 (0.046) | 0.014 (0.076) | 0.007 (0.030) | 0.025 (0.083) | 0.013 (0.022) | 0.039 (0.053) |
|  | BAT | 0.016 (0.043) | 0.014 (0.038) | 0.019 (0.046) | 0.042 (0.100) | 0.017 (0.022) | 0.021 (0.020) |
|  | Active enforcer | 0.013 (0.051) | 0.016 (0.058) | 0.012 (0.036) | 0.037 (0.062) | 0.017 (0.023) | 0.039 (0.061) |
|  | Inactive enforcer | 0.027 (0.061) | 0.015 (0.049) | 0.008 (0.032) | 0.010 (0.112) | 0.020 (0.025) | 0.036 (0.052) |
|  | Total | 0.016 (0.051) | 0.015 (0.057) | 0.011 (0.036) | 0.029 (0.090) | 0.017 (0.023) | 0.034 (0.049) |
| Levator | No exposure | 0.004 (0.037) | 0.020 (0.030) | 0.010 (0.021) | 0.027 (0.041) | 0.009 (0.014) | 0.024 (0.040) |
|  | BAT | 0.015 (0.023) | 0.016 (0.028) | 0.021 (0.024) | 0.030 (0.042) | 0.011 (0.017) | 0.017 (0.024) |
|  | Active enforcer | 0.003 (0.035) | 0.014 (0.029) | 0.011 (0.022) | 0.025 (0.030) | 0.012 (0.015) | 0.014 (0.020) |
|  | Inactive enforcer | 0.017 (0.029) | 0.019 (0.038) | 0.018 (0.022) | 0.034 (0.038) | 0.007 (0.014) | 0.016 (0.033) |
|  | Total | 0.009 (0.032) | 0.017 (0.031) | 0.015 (0.023) | 0.029 (0.038) | 0.010 (0.015) | 0.018 (0.030) |

Table S2: Title S2. Muscular response per Phase per CS

Table S2. Legend S2. Mean EMG activity in microvolt for muscle type (corrugator, levator) as a function of phase (habituation, acquisition, extinction) and type of CS per group
