# Supplementary material for: Optimising Extinction of Conditioned Disgust
Source: PLoS One. 2016 Feb 5;11(2):e0148626. doi: 10.1371/journal.pone.0148626 (PMC4743916; doi:10.1371/journal.pone.0148626)
Supplement: S1 Appendix — (DOCX) [file pone.0148626.s003.docx]

**S3 Appendix**

**Manipulation checks for the conditioned stimuli (CS)**

Three separate T-tests were conducted to test for pre-existing differences on *disgust, willingness-to-eat,* and *valence*. This analysis was based on the habituation scores, with means collapse over both images of each category. The analysis showed that the sausage roll [M = 25.9, SD = 23.9] was evaluated as less disgusting than the soup [M = 43.3, SD = 28.1; *t* (530) = 7.7, p < 0.001]. Also the willingness-to-eat was higher for the sausage roll [M = 53.9, SD = 27.0] than for the soup [M = 40.7, SD = 26.5; *t* (530) = 5.7, p < 0.001]. Lastly, the sausage roll [M = 56.5, SD = 21.9] was evaluated more positively than the soup [M = 41.9, SD = 23.9; *t* (530) = 7.3, p < 0.001]. These ratings were not in line with what was expected based on the validation, however they were not considered problematic due to the counterbalanced design of the experiment.
